# Association between the cardiometabolic index and chronic kidney disease: a cross-sectional study

**DOI:** 10.1007/s11255-023-03888-4

**Published:** 2023-12-08

**Authors:** Qian Guo, Yani Wang, Yuchen Liu, Yun Wang, Lin Deng, Lihua Liao, Xueqin Lin, Mingxin Wu, Meirui Sun, Ying Liao

**Affiliations:** 1 Department of Cardiology, Longyan First Affiliated Hospital of Fujian Medical University, Longyan, 364000 China; 2 Department of Electrocardiography, Longyan First Affiliated Hospital of Fujian Medical University, Longyan, 364000 China

**Keywords:** Cardiometabolic index, Central obesity, Chronic kidney disease, NHANES

## Abstract

**Background:**

Central obesity is a risk factor for chronic kidney disease (CKD). However, the exact correlation between the cardiometabolic index (CMI), an indicator of central obesity, and CKD remains unclear. Here, we aimed to investigate the correlation between the CMI and CKD in the general American population.

**Methods:**

This cross-sectional study involved 64,313 members of the general population (≥ 20 years of age) with data in the National Health and Nutrition Examination Survey (NHANES) 1999–2020. The individuals were grouped into three categories by CMI tertile: T1 group (n = 7,029), T2 group (n = 7,356), and T3 group (n = 7,380). Logistic regression analysis was performed, with NHANES recommended weights, to assess the association between the CMI and CKD.

**Results:**

A total of 21,765 participants were included; the overall prevalence of CKD was 12.2%. From the low to the high CMI tertile, the prevalence of CKD increased from 8.9% to 16.0% (P < 0.001). After full adjustment for confounders, the higher tertile of CMI (OR: 1.08, 95% CI: 1.03 − 1.13, P = 0.002) had the higher risk of CKD. Compared with the T1 group, the groups with higher CMI levels had a higher CKD risk (T2: OR: 1.01, 95%CI: 0.87–1.18, P = 0.812; T3: OR: 1.22, 95%CI: 1.05–1.43, P = 0.013).

**Conclusions:**

Higher CMI was independently associated with higher CKD risk in the general population.

## Introduction

Chronic kidney disease (CKD) is an irreversible and progressive condition [1] that is emerging as a serious and widely recognized public health issue [2]. More than 850 million individuals have been estimated to have kidney disease worldwide [3]. To mitigate the disease burden, CKD must be prevented in the general population.

Central obesity is strongly associated with CKD [4]. Moreover, central fat distribution has been associated with diminished estimated glomerular filtration rate (eGFR) and effective renal plasma flow, and elevated glomerular hyperfiltration [5]. The cardiometabolic index (CMI), an indicator used to assess central obesity, comprises the waist-to-height ratio (WHtR), and triglyceride (TG) and high-density lipoprotein cholesterol (HDL-C), and was first developed by Wakabayashi et al. in 2015 [6]. In the past several years, studies have focused on association of the clinical CMI value with atherosclerosis, hypertension, ischemic stroke, left ventricular dilation, diabetes, and fatty liver disease, and CMI has been found to perform well in assessing the emergence and advancement of these illnesses [6–11]. However, few studies have investigated the relationship between CMI and CKD, which remains unclear.

Therefore, our study aim was to explore the relationship between CMI and CKD in the general American population, to provide insights into CKD prevention and control.

## Methods

### Study design and population

**Fig. 1:**
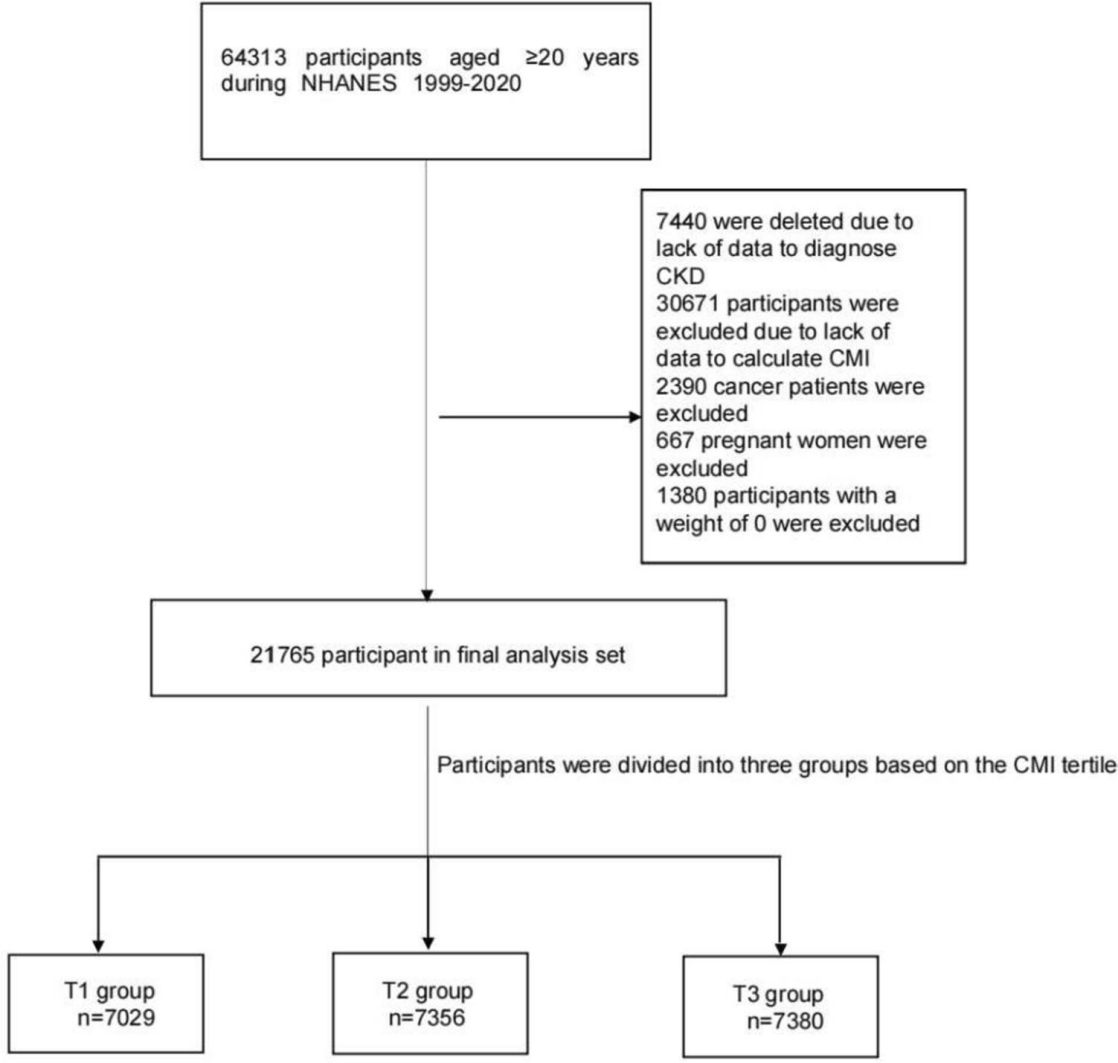
Flowchart of the study design

The objective of the National Health and Nutrition Examination Survey (NHANES) is to comprehensively assess the health and nutrition of individuals across all age groups in the United States. Using a complex weighted analysis, the survey ensures representation of the entire United States population by sampling approximately 5,000 individuals from different counties across the country every 2 years. We identified 64,313 individuals from United States communities who voluntarily participated in the NHANES survey between 1999 and 2020, and were above 20 years of age. Of these, 7,440 were excluded from the analysis because of insufficient data for diagnosing CKD, and 30,671 were excluded because of a lack of data for calculating the CMI. Furthermore, 2,390 individuals with a history of cancer, 667 pregnant people, and 1,380 people with a sampling weight of 0 were also excluded from the analysis. Thus, the analysis herein was based on 21,765 included individuals (Fig. 1).

### Calculation of the CMI

The WHtR refers to the waist circumference in centimeters divided by the height in centimeters. The TG/HDL-C ratio represents the relationship between TG and HDL-C levels. The WHtR is multiplied by TG/HDL-C to calculate the CMI. On the basis of CMI tertiles, the participants were categorized into three groups: T1 group (CMI ≤ 0.8), T2 group (0.8 < CMI ≤ 1.7), and T3 group (CMI > 1.7).

### Primary outcome

The primary endpoint was CKD. As recommended by Kidney Disease: Improving Global Outcomes (KDIGO), CKD is defined by a consistently low eGFR below 60 mL per minute per 1.73 m^2^ body surface area, or excretion of ≥ 30 mg urinary albumin per day [12].

### Definitions of variables of interest

Participants provided self-reported information on their age, gender, ethnicity, smoking habits, and alcohol consumption. A physical examination was conducted to obtain waist and height measurements. Individuals whose body mass index (BMI) exceeded 30 were considered obese. Diabetes mellitus was characterized by either a self-reported diabetes history or a fasting blood glucose level ≥ 7.0 mmol/L documented during the survey. Individuals were categorized as having hypertension if they had been previously diagnosed with hypertension or if their blood pressure measurements during the field survey indicated a resting systolic blood pressure of 130 mmHg or higher and/or a diastolic blood pressure of 80 mmHg or higher. Automated hematological analysis equipment was used to collect laboratory measurements, including total cholesterol (TC), TG, low-density lipoprotein cholesterol (LDL-C), and HDL-C.

### Statistical analyses

The calculation of weights for specific groups was performed according to the NHANES recommended weights. Since NHANES is a sampling design and needs to reflect the error between the sample and the whole in terms of the standard error, the baseline characteristics are to be presented in terms of the mean (standard error). Whereas counts (and percentages) were used to present categorical variables. Analysis of variance was used to compare the baseline characteristics among groups in terms of continuous variables, whereas a χ2 test was used for categorical variables. To evaluate the link between CMI and CKD, we performed logistic regression analyses. According to the recommended weights from NHANES, complex sampling weighted analysis was used for all statistical analyses. To improve the reliability of the findings, we examined three models. The first model, model 1, was the unadjusted model. Model 2 included adjustment for age, gender, and race, whereas model 3 included comprehensive adjustment for potential confounding factors such as age, gender, race, smoking habits, alcohol consumption behaviors, BMI, TC, LDL-C, hypertension, and diabetes mellitus (DM). To analyze the possible nonlinear correlation between CMI and CKD, we also used regression cubic spline (RCS) analysis. The RCS was adjusted for the same variables as those in model 3, to ensure consistency. Furthermore, we performed a stratified analysis according to age, gender, obesity, smoking, alcohol consumption, hypertension, and diabetes, and investigated the potential interplay between CMI and these subsets. The Survey package in R software was used for data analyses (version 4.2.2; R Foundation for Statistical Computing, Vienna, Austria). For all analyses, a significance threshold < 0.05 was considered statistically significant.

## Results

### Participant characteristics

Table 1 presents the baseline clinical characteristics. In this study, 21,765 participants were included, with an average age of 45.5 (0.2) years, 10,825 (49.9%) of whom were men. In total, 3,521 (12.2%) had CKD, 10,759 (45.2%) had hypertension, and 3,974 (13.2%) had DM. Participants were divided into three groups by CMI tertile [T1 (n = 7,029); T2 (n = 7,356); and T3 (n = 7,380)]. Compared with the T1 group, the T3 group had higher LDL-C [T1: 104.09 mg/dl vs. T3: 122.31 mg/dl, P < 0.001], TG [T1: 63.10 mg/dl vs. T3: 216.26 mg/dl, P < 0.001], body obesity [T1: 13.5% vs. T3: 56.9%, P < 0.001], and WHtR [T1: 0.52 vs. T3: 0.64, P < 0.001]. Furthermore, groups with higher CMI had higher proportions of hypertension [T1: 30.8% vs. T2: 45.5% vs. T3: 59.4%, P < 0.001] and DM [T1: 5.2% vs. T2: 11.7% vs T3: 22.7%, P < 0.001]. Additional comprehensive data are provided in Table 1.

**Table 1:** Baseline study population characteristics (weighted)

Variable	Total (N = 21,765)	T1 Group (N = 7029)	T2 Group (N = 7356)	T3 Group (N = 7380)	*P*-value
Age	45.5 (0.2)	42.5 (0.3)	46.2 (0.3)	47.8 (0.3)	< 0.001
Male, n (%)	10,825 (49.9)	2841 (39.5)	3621 (49.2)	4363 (60.9)	< 0.001
Race, n (%)					< 0.001
Mexican American	3949 (8.8)	811 (6.3)	1348 (9.1)	1790 (11.1)	
Non-hispanic black	4529 (11.5)	2072 (16.1)	1597 (11.8)	860 (6.5)	
Non-hispanic white	8863 (66.2)	2680 (64.5)	2920 (65.2)	3263 (69.0)	
Other hispanic	2015 (6.0)	526 (5.0)	727 (6.5)	762 (6.4)	
Other race	2409 (7.5)	940 (8.2)	764 (7.3)	705 (7.0)	
TC, mg/dl	193.84 (0.46)	183.10 (0.63)	193.52 (0.68)	204.93 (0.73)	< 0.001
TG, mg/dl	128.27 (1.16)	63.10 (0.34)	105.43 (0.49)	216.26 (2.47)	< 0.001
LDL-C, mg/dl	115.37 (0.37)	104.09 (0.54)	120.09 (0.55)	122.31 (0.58)	< 0.001
HDL-C, mg/dl	53.40 (0.19)	66.40 (0.29)	52.33 (0.20)	41.47 (0.15)	< 0.001
TG/HDL-C	2.89 (0.03)	0.99 (0.01)	2.05 (0.01)	5.65 (0.08)	< 0.001
Waist, cm	98.36 (0.21)	87.80 (0.21)	98.91 (0.24)	108.36 (0.31)	< 0.001
Height, cm	169.14 (0.10)	168.34 (0.16)	168.74 (0.16)	170.32 (0.16)	< 0.001
WHtR	0.58 (0.00)	0.52 (0.00)	0.59 (0.00)	0.64 (0.00)	< 0.001
CMI	1.67 (0.02)	0.52 (0.003)	1.18 (0.01)	3.32 (0.03)	< 0.001
Obese, n (%)	8046 (35.5)	1176 (13.5)	2782 (36.2)	4088 (56.9)	< 0.001
Smoke, n (%)	9686 (45.7)	2676 (39.4)	3225 (45.0)	3785 (52.6)	< 0.001
Drink, n (%)	14,108 (76.8)	4836 (81.3)	4703 (76.4)	4569 (72.7)	< 0.001
DM, n (%)	3974 (13.2)	599 (5.2)	1278 (11.7)	2097 (22.7)	< 0.001
Hypertension, n (%)	10,759 (45.2)	2,572 (30.8)	3,706 (45.5)	4,481 (59.4)	< 0.001
CKD, n (%)	3521 (12.2)	817 (8.9)	1166 (11.8)	1538 (16.0)	< 0.001

Values are, n (%) or mean (SE)

*TG* triglyceride, *TC* total cholesterol, *HDL*-*C* high-density lipoprotein cholesterol, *LDL*-*C* low-density lipoprotein cholesterol, *WHtR* waist-to-height ratio, *CMI* cardiometabolic index, *DM* diabetes mellitus, *CKD* chronic kidney disease

### Association between CMI and CKD

**Fig. 2:**
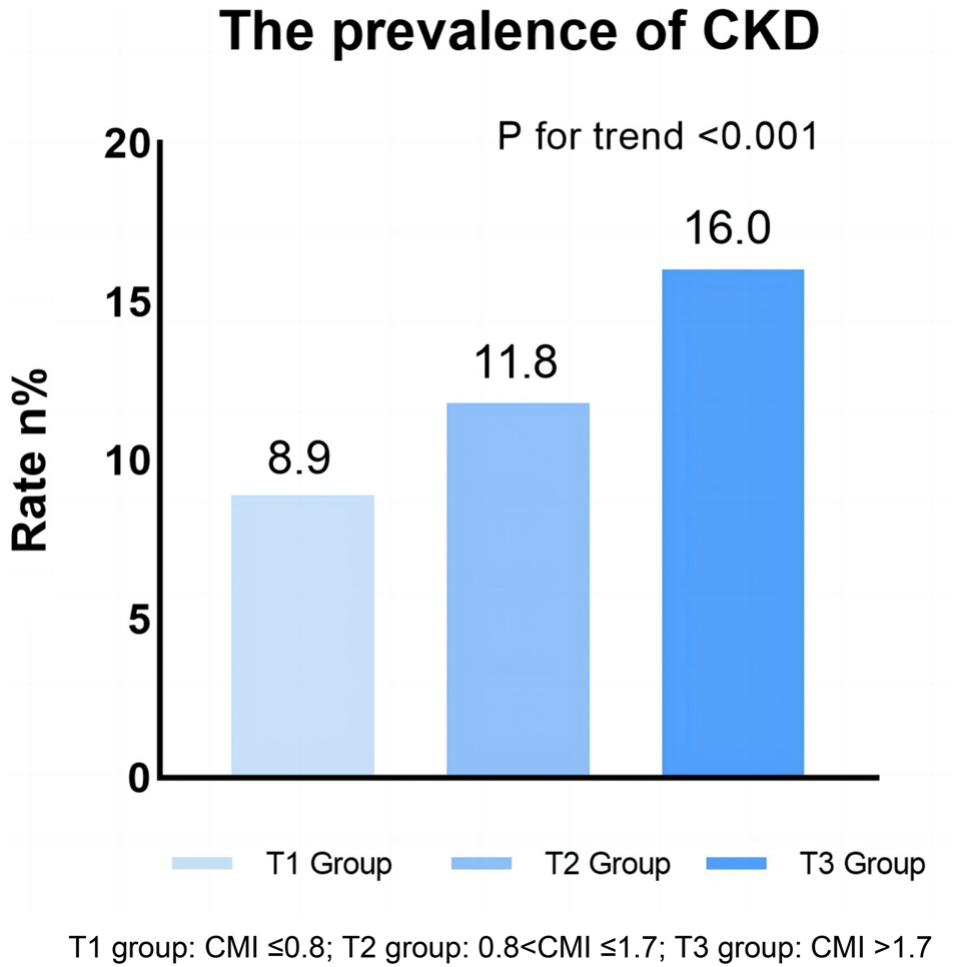
Prevalence of CKD in different groups (weighted)

With increasing CMI, the incidence rate of CKD gradually increased [T1: 8.9% vs. T2: 11.8% vs. T3: 16.0%, P < 0.001] (Fig. 2). Univariate logistic regression analysis indicated that CMI was positively correlated with CKD in the participants (OR: 1.15, 95% CI: 1.12 − 1.18, P < 0.001). Compared with the T1 group, the groups with higher CMI had a higher risk of CKD (T2: OR: 1.36, 95% CI: 1.21–1.53, P < 0.001; T3: OR: 1.95, 95% CI: 1.71–2.21, P < 0.001). After adjustment for age, gender, race, smoking status, alcohol consumption status, BMI, TC, LDL-C, hypertension, and DM, the association between CMI (OR: 1.08, 95% CI: 1.03 − 1.13, P = 0.002) and CKD did not change. Moreover, the groups with higher CMI also had a higher risk of CKD (T2: OR: 1.01, 95% CI: 0.87–1.18, P = 0.812; T3: OR: 1.22, 95% CI: 1.05–1.43, P = 0.013) (Table 2).

**Table 2:** The association between CMI and CKD (weighted)

Variable		Model 1		Model 2		Model 3	
		OR (95% CI)	P-value	OR (95% CI)	P-value	OR (95% CI)	P-value
Continuous variables							
CMI per 1 unit		1.15 (1.12–1.18)	< 0.001	1.17 (1.14–1.21)	< 0.001	1.08 (1.03–1.13)	0.002
CMI per 1 SD		1.38 (1.30–1.47)	< 0.001	1.45 (1.35–1.56)	< 0.001	1.20 (1.07–1.34)	0.002
Categorical variable	Event/All population						
T1 Group	817/7029	Ref		Ref		Ref	
T2 Group	1166/7356	1.36 (1.21–1.53)	< 0.001	1.18 (1.03–1.35)	0.016	1.01 (0.87–1.18)	0.812
T3 Group	1538/7380	1.95 (1.71–2.21)	< 0.001	1.76 (1.53–2.03)	< 0.001	1.22 (1.05–1.43)	0.013

Model 1: Not adjusted

Model 2: Adjusted by age, gender, race/ethnicity

Model 3: Adjusted by age, gender, race/ethnicity, smoke, drink, BMI, TC, LDL-C, hypertension and DM

### Subgroup analysis

**Fig. 3:**
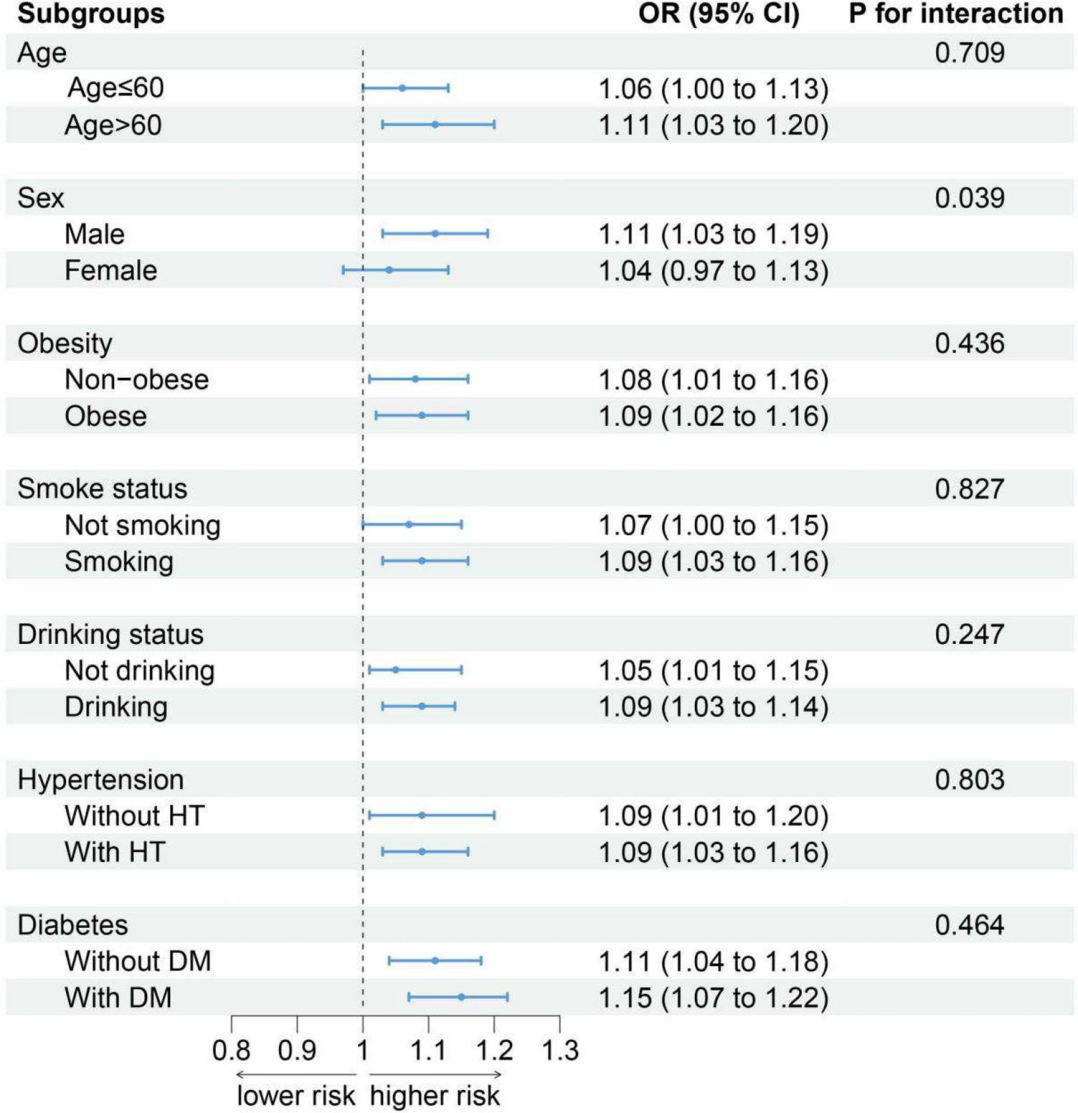
Association between CMI and CKD by selected subgroups (weighted)

After stratification of participants by age (P for interaction = 0.709), obesity (P for interaction = 0.436), smoking status (P for interaction = 0.827), alcohol consumption status (P for interaction = 0.247), hypertension (P for interaction = 0.803), diabetes (P for interaction = 0.464), and sex [male (OR: 1.11, 95% CI: 1.03 − 1.19); female (OR: 1.04, 95% CI: 0.97 − 1.13), P for interaction = 0.039], the only observed interaction was between gender and CMI. Men, but not women, showed an association of CKD with CMI. Compared with the T1 group, groups with higher CMI had a greater risk of developing CKD (Fig. 3).

### Regression cubic splines

**Fig. 4:**
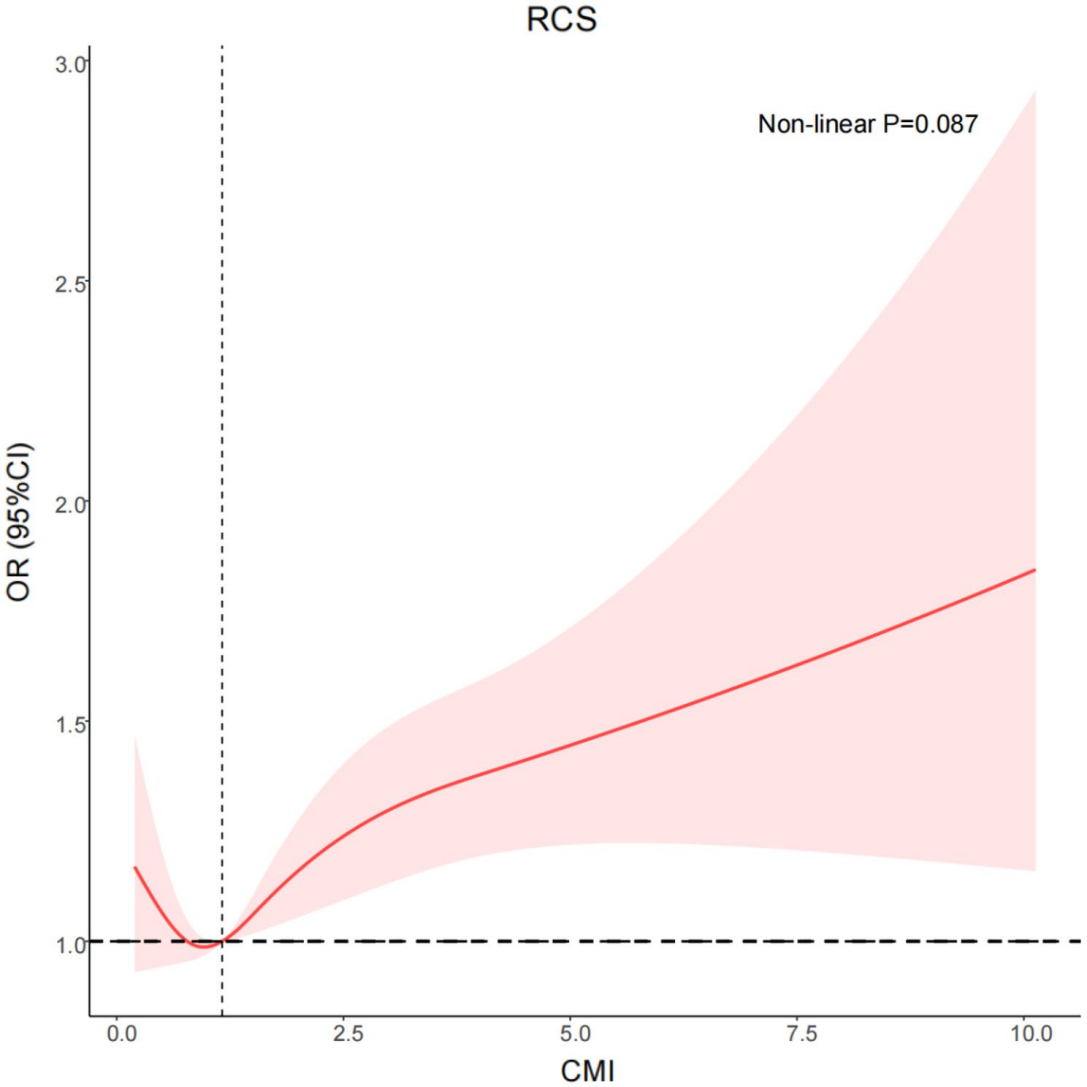
Potential nonlinear relationship between CMI and CKD (weighted)

RCS indicated a J-shaped relationship between CMI and CKD. Elevated CMI in participants was associated with higher risk of developing CKD. A similar trend was observed in the relationship between extremely low CMI and CKD (non-linear P = 0.087) (Fig. 4).

## Discussion

In this cross-sectional study, the CMI was associated with CKD risk, such that higher CMI was associated with greater CKD risk. We observed no significant change in the association between CMI and CKD in the general American population after stratification according to age, obesity, smoking status and alcohol consumption status, hypertension, and diabetes. In the analysis of sex subgroups, we found that CMI was not significantly correlated with CKD in women.

CMI, a measure of central obesity, has been used to assess the occurrence of atherosclerosis, fatty liver associated with metabolism, and hyperuricemia, with promising results [7, 11, 13]. However, relatively less research has been conducted on the association between CMI and CKD, although CKD has been correlated with central obesity [14, 15].

To our knowledge, only two previous studies have examined the association between CMI and the development of CKD [16, 17]. First, a research project in rural areas of China has described the utility of considering diminished eGFR with CMI [16]. Chinese and American populations differ in blood lipids and body types: Misra et al. have reported that Asian populations exhibit distinct body composition characteristics characterized by greater abdominal adiposity than observed in numerous other ethnic groups [18]. Therefore further studies are needed to determine whether this conclusion applies to the United States population, given that a previous study has suggested that CMI was negatively associated with kidney function in the Asian population. Moreover, another study has found that CMI was negatively correlated with renal function in people older than 60 years in America [17]. Nevertheless, because the incidence of CKD is increasingly affecting younger people, exploring only older people is inadequate [19–21]. To our knowledge, this study is the first to explore the association of CMI with CKD in the general American population.

Although a potential causative mechanism between CMI and CKD remains unclear, our findings are supported by previous research. Hou et al. have identified a positive association between TG level and diminished eGFR [22]. TG catabolism is delayed by downregulation of hydrolysis of very low density lipoproteins and chylomicrons by lipoprotein lipase, thus leading to lipid accumulation in renal tissues [23], and consequently inflammation, oxidative stress, and autophagy. Ultimately, substantial proliferation of glomerular basement membrane cells occurs, thus exacerbating glomerulosclerosis and tubulointerstitial injury, and promoting the onset of proteinuria [21, 24, 25]. Tozawa et al. have demonstrated that HDL-C was positively associated with eGFR [26]. Moreover, Ho et al. have demonstrated that the TG/HDL-C ratio was an independent risk factor for CKD [27]. The role of obesity in the occurrence of focal segmental glomerulosclerosis and glomerulomegaly has been examined in previous studies [28, 29]. A population-based study has suggested global use of WHtR to assess CKD across ethnic groups [30]. Odagiri et al. have identified the WHtR as a stand-alone factor that could reliably predict the incidence of CKD [31]. These findings have established a theoretical foundation for the prevention of CKD through the regulation of CMI levels.

In subgroup analyses stratified by age, obese, smoking status, alcohol consumption status, hypertension, and diabetes, the outcome of the regression analysis did not show any significant alteration. Elevated CMI was an independent risk factor for CKD. Furthermore, an interaction between CMI and gender was observed. We speculated that this interaction might have arisen from differences in hormones between sexes and a protective effect of estrogen in inhibiting renal injury induced by dyslipidemia [32–35]. We further investigated a potential nonlinear correlation between CMI and CKD with restricted RCS analysis. No evidence of a potential non-linear relationship was detected in the RCS data, which indicated a J-shaped relationship. Very low CMI appeared to have no notable protective influence on participants with CKD. These findings might be explained as follows. The CMI was calculated as TG/HDL-C ratio × WHtR [6]. Patients with very low CMI also have low level of lipids and WHtR, thus potentially leading to malnutrition or cachexia [36, 37], thereby increasing CKD risk [38–40]. Thus, individuals with very low CMI might have faced an elevated risk of malnutrition that obscured the advantages of very low CMI. However, future studies are necessary to validate this hypothesis.

This study had several limitations. First, due to the inherent limitations of cross-sectional analysis. The link between CMI and CKD could be perceived only as correlative rather than causative. Second, as with other cross-sectional studies, our study might have been susceptible to the influences of additional variables that could have confounded the results. Third, subject to the limitations of public databases, there was no information on kidney transplantation in NHANES, and the impact of kidney transplantation needs to be further explored in future prospective studies. Finally, additional longitudinal follow-up studies are necessary to validate whether CMI might serve as an early screening tool for the prevention of CKD in patients.

## Conclusion

CMI was positively associated with CKD risk. We advocate for the widespread implementation of CMI to evaluate potential CKD risk in the general population. Moreover, our study found that the contribution of very low CMI to CKD prevention may be limited.

## Data Availability

Publicly available datasets were analyzed in this study. This data can be found in NHANES’s official website, at http://www.cdc.gov/nchs/nhanes.htm.

## Abbreviations

CKD: Chronic kidney disease
eGFR: Estimated glomerular filtration rate
CMI: Cardiometabolic index
WHtR: Waist-to-height ratio
TG: Triglyceride
HDL-C: High-Density Lipoprotein Cholesterol
NHANES: National Health and Nutrition Examination Survey
KDIGO: Kidney Disease: Improving Global Outcomes
BMI: Body mass index
TC: Total cholesterol
LDL-C: Low-Density Lipoprotein Cholesterol
DM: Diabetes mellitus
RCS: Regression cubic spline
